# Multiple *b*-values improve discrimination of cortical gray matter regions using diffusion MRI: an experimental validation with a data-driven approach

**DOI:** 10.1007/s10334-021-00914-3

**Published:** 2021-03-12

**Authors:** Tara Ganepola, Yoojin Lee, Daniel C. Alexander, Martin I. Sereno, Zoltan Nagy

**Affiliations:** 1 Department of Cognitive, Perceptual and Brain Sciences, University College London, London, UK; 2 Center for Medical Image Computing, Department of Computer Science, University College London, London, UK; 3 Laboratory for Social and Neural Systems Research, University of Zurich, Rämistrasse 100, P.O. Box 149, Zurich, Switzerland; 4 Institute of Biomedical Engineering, ETH Zurich, Zurich, Switzerland; 5 Department of Psychology and Neuroimaging Centre, SDSU, San Diego, USA; 6 Wellcome Trust Centre for Neuroimaging, UCL Institute of Neurology, London, UK

**Keywords:** Cortical parcellation, Brodmann map, In-vivo histology, Diffusion MRI, Microstructural imaging

## Abstract

**Objective:**

To investigate whether varied or repeated *b*-values provide better diffusion MRI data for discriminating cortical areas with a data-driven approach.

**Methods:**

Data were acquired from three volunteers at 1.5T with *b*-values of 800, 1400, 2000 s/mm^2^ along 64 diffusion-encoding directions. The diffusion signal was sampled from gray matter in seven regions of interest (ROIs). Rotational invariants of the local diffusion profile were extracted as features that characterize local tissue properties. Random forest classification experiments assessed whether classification accuracy improved when data with multiple *b*-values were used over repeated acquisition of the same (1400 s/mm^2^) *b*-value to compare all possible pairs of the seven ROIs. Three data sets from the Human Connectome Project were subjected to similar processing and analysis pipelines in eight ROIs.

**Results:**

Three different *b*-values showed an average improvement in correct classification rates of 5.6% and 4.6%, respectively, in the local and HCP data over repeated measurements of the same *b*-value. The improvement in correct classification rate reached as high as 16% for individual binary classification experiments between two ROIs. Often using only two of the available three *b*-values were adequate to make such an improvement in classification rates.

**Conclusion:**

Acquisitions with varying *b*-values are more suitable for discriminating cortical areas.

## Introduction

In-vivo cortical parcellation aims to non-invasively differentiate the cortical areas that have been shown to contain distinctly specialized layering of gray matter (GM) tissue [1–5]. Although a one-to-one correspondence cannot be expected between structure and function [6, 7], tissue differentiation in GM has been related in both animals [4] and humans [8] to different functional roles and is, by now, widely accepted [9–11]. Because functional mapping of the entire cortex requires a prohibitively large number of task-based fMRI experiments to be daily practice [12], the possibility of relying on structural MR images of a single scanning session for the in-vivo parcellation of the cortex is particularly attractive [13, 14].

The diffusion constant [15], T1- and T2 relaxation times [16, 17], magnetization transfer [18], etc. are each thought to be sensitive to some aspects of the underlying tissue microstructure [19, 20]. Importantly, quantitative MRI data, may be used in two related albeit vastly different approaches. On the one hand, one may wish to parcellate the cortex based on histologically relevant variables (e.g. myelination, iron content or cell size) that are obtained from invertible biophysical models [13, 14]. On the other hand, a fingerprint that is obtained directly from the quantitative measurements can be used to classify tissue without constructing such a model. Importantly, we chose the latter, data-driven, approach and employed high angular resolution diffusion imaging (HARDI) [21, 22] because, unlike other contrast mechanisms, which provide a single degree of freedom, a HARDI acquisition probes the same tissue multiple times in varying spatial directions to provide a rich data set. Such data have been shown to provide ample information to characterize GM tissue and thus identify different cortical areas [23] or layers [24–26].

The ultimate aim of such parcellation methods is a non-invasive, reproducible and anatomically reliable parcellation of the in-vivo human cortex. In our previous effort toward this aim, we put forth a model-free approach [27]. While the reliability of this proof of principle we validated with a test/re-test approach, several avenues for improvement were also outlined in that early work. Subsequently, we tested various feature sets that are extractable from HARDI data [28], and obtained preliminary evidence that indicated a preference for including multiple *b*-values in the HARDI data for classification of GM tissue [28, 29]. Although the latter supposition is reasonable, a systematic controlled experiment has not confirmed it. Accordingly, the aim of the present study was to systematically and empirically ascertain that multiple different *b*-values improve discrimination between cortical areas [27].

## Methods

All analyses aimed at comparing the useful information content in data that contained two or three acquisitions of the same *b*-value against data that also included two or three acquisitions, but with different *b*-values. Although, repeated acquisitions of the same *b-value* is an often-used technique to improve SNR [30–36], the main motivation here was to avoid confounds that would otherwise result from comparing cases with variable data sizes. In all comparisons between the repeated or mixed *b*-values the amount of data were identical (i.e. the three acquisitions of the same *b-value* were never averaged).

### Imaging data

#### Local 1.5T data

Data were collected from three healthy adult male volunteers on a 1.5T Siemens Avanto scanner and a 32-channel head coil (Siemens Healthcare, Erlangen Germany) with approval from the National Hospital for Neurology and Neurosurgery and Institute of Neurology Joint Research Ethics Committee and signed written informed consent from each participant. Three *b*-values were sampled in ten HARDI data sets with four reference images (*b*_o_ = 0 s/mm^2^) and 64 diffusion-weighted images (DWIs), multiband factor of 2, spatial resolution of 1.7 × 1.7 × 1.7 mm^3^. Each HARDI data set took 6.8 min. The acquisition order was *b* = 1400, 800, 1400, 2000, 1400 s/mm^2^ with TE/TR = 86/5647, 94/5980, 101/6224 ms for the increasing *b*-values and each collected with both blip-up/down phase encoding. All ten HARDI data sets were simultaneously fed through *topup* and *Eddy* [37] in FSL release 5.0.9 to align them and curtail susceptibility- and eddy-current-induced distortions. The diffusion directions were not varied among the repeated acquisition of the data with *b* = 1400 s/mm^2^. T1-weighted (T1w) MPRAGE images were also acquired in 2.8 min with 1.0 × 1.0 × 2.0 mm^3^ voxels, TE/TI/TR = 4/1000/1370 ms and twofold mSENSE phase encoding acceleration.

#### HCP 3T data

The HARDI data of three subjects from the Human Connectome Project (HCP) 500-subject data release [38–40] were from a 3T Siemens Skyra system with a 100 mT/m gradient coil, across three interleaved b-shells (*b* = 1000, 2000, 3000 s/mm^2^) at an isotropic spatial resolution of 1.25 × 1.25 × 1.25 mm^3^ and TE/TR of 89.5/5520 ms. The *b*-values were modulated by varying *G*_max_ with 18 *b*_o_ images and contained 90 diffusion directions in each b-shell [41]. Because the HCP data did not contain repeated acquisitions of any of the *b*-values (and in particular not the middle *b*-value of 2000 s/mm^2^) a different approach was set up that enabled the subsequent analysis steps to be identical for the 3T and 1.5T data. In this approach, the DWIs from each b-shell were split via an electrostatic repulsion algorithm [42] into three subsets. Each of the subsets contained 30 evenly spaced diffusion directions. This step provided data that could be considered three repeats of the same *b-value* for all three *b-value* shells. To synthesize data with repeated *b-value* of 2000 s/mm^2^, each subset with 30 diffusion direction was processed separately to extract 9 features (described below in detail) and the features concatenated. To synthesize data with different *b*-values, one of the three subsets with 30 diffusion-encoding directions was chosen from each of the three *b-value* shells. The T1w images with isotropic spatial resolution of 0.7 × 0.7 × 0.7 mm^3^ were also saved.

### Surface-based image processing pipeline

#### Sampling to the cortical surface

To extract feature vectors [27, 28], the T1w image was used to generate a boundary surface between the GM and white matter (WM) for each subject using FreeSurfer 5.3 [43] *recon-all*. The *b*_o_ image (or mean of the 18 *b*_o_ images in HCP data) was registered to the T1w volume using manual blink comparison and affine transformation (*tkregister* in *csurf*) and the registration matrix was applied to the DWIs. For each vertex of the surface tessellation, the voxel that contained the 50% point of the local cortical thickness estimate (FreeSurfer 5.3 *mris_thickness*) outward from the GM/WM surface along the local surface normal was sampled from each diffusion direction data set (*paint* in *csurf*).

#### Feature representation

A 6th order spherical harmonic series was fit to the extracted cortical HARDI data in each b-shell [27] from which a smaller set of nine features were generated as in Ganepola et al. [28]. Four of these features are fully rotationally invariant, while the remaining five are invariant relative to the local normal vector to the GM/WM boundary surface. When combining data sets these feature vectors were concatenated, resulting in either a 1 × 18 or a 1 × 27 feature vector at each vertex [44] for the comparisons with two or three repeats respectively of the same vs. different *b*-values.

### Data analysis

#### Correlation maps

Correlation coefficients between the feature vectors of each HARDI data set [29] served as a simple test of our hypothesis in that high/low correlation was expected between vectors from identical/different *b *-values, respectively. The correlation coefficient between data sets with the same *b-value* also served as a surrogate marker of data quality, because high correlation indicates repeatable experiments.

#### Regions of interest

Most regions of interests (ROIs) used in this study were related to Brodmann areas [1, 3]. For the 1.5T data, seven ROIs were defined. Areas 3b (primary somatosensory), 4p (primary motor), and 45 (Broca’s area) were delineated with FreeSurfer Brodmann Atlas, while the middle temporal area (MT), ventral intraparietal (VIP) [45], a section of the angular gyrus (AnG) within the default mode network and Insular (a small region in the auditory core in the lateral sulcus) were defined in a previous study using multimodal data [27].

HCP ROIs were defined using the HCP Multi-Modal Parcellation (HCP-MMP) [46]. Eight labels were selected and registered from the *fsaverage* surface to the subject-specific surface tessellations using surface based registration [47]. Some of the ROIs were equivalent to those above (3b, 4, MT and A1). The remaining regions were V1, V2, FST, and Lbelt, corresponding to the primary and secondary visual areas, the fundus of superior temporal sulcus and the lateral auditory belt region, respectively.

**Fig. 1:**
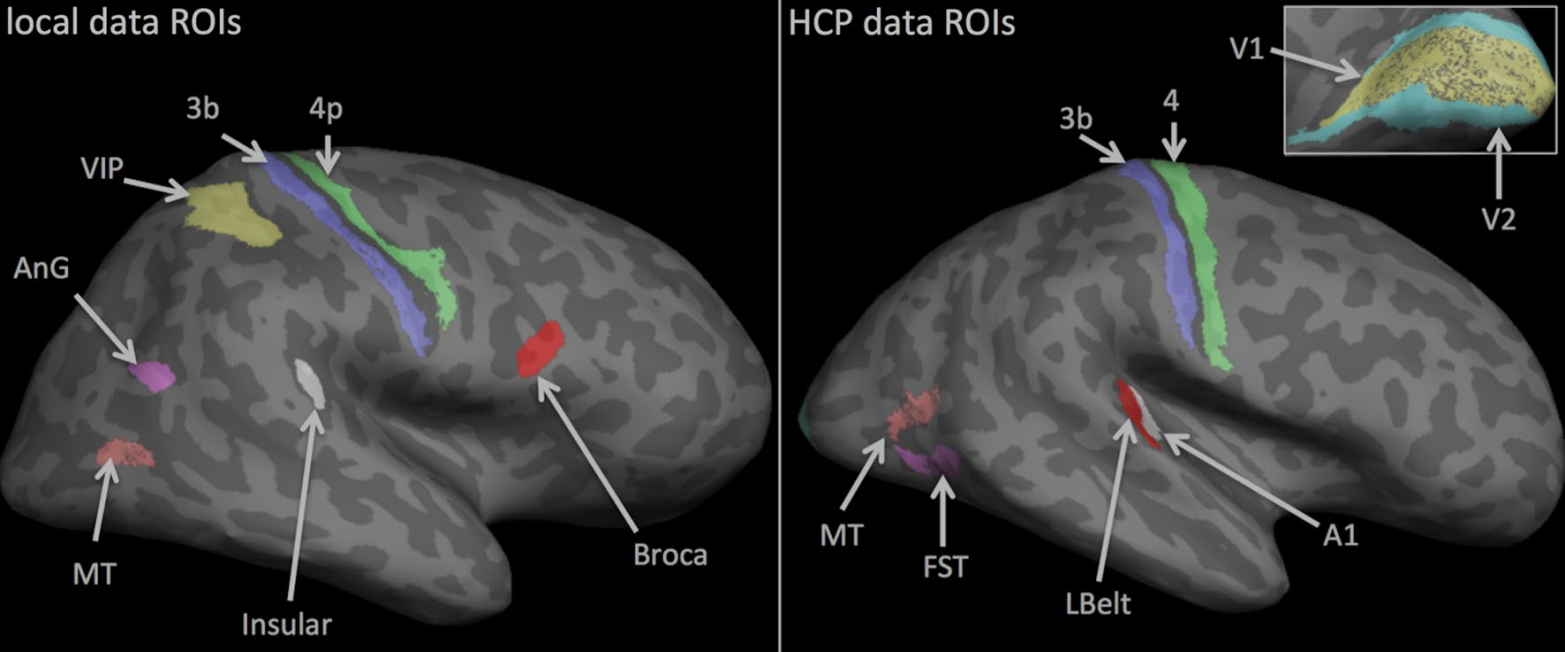
Pictorial depiction of the ROIs used for the binary classification experiments for both the local (left) and the HCP (right) data. See text for further details

The two different sets of ROIs (Fig. 1) were selected to encompass a wide range of cortical tissue types within each dataset and even wider range when the results of both datasets are combined, which help ensure robustness of the processing steps and classification results.

#### Classification experiments

All 21/28 binary combinations of these seven/eight ROIs for the 1.5T and 3T data were tested in separate binary classification experiments for each subject and both the mixed and repeated *b-value* combinations. The random forest classification [48] used *sklearn* with *n_estimators* = 15 and default values on the other hyperparameters. Test/train data sets were generated using leave-one-out cross validation, similar to that in Ganepola et al. [28], in which the classifier is trained on the feature vectors from all but one of cortical vertex points in the two ROIs, tested on the feature vector from the unseen vertex and repeating the process until all vertices in the two ROIs were tested. Because the resolution of the MPRAGE data was higher, multiple vertices could sample the same HARDI voxel. To avoid duplication between the test and training data, the number of vertices for each ROI were reduced to a set that provided a one-to-one mapping using only the vertex closest to the average location of vertices that sampled the same voxel (*tksurfer* in *csurf*).

In addition to comparing the performance with three different *b*-values against the data collected three times with identical *b*-value, further comparisons were made on partial data sets that contained only two acquisitions: either different or repeated *b*-values. Namely, binary classification experiments were performed on features extracted from the (a) lowest and middle *b*-values, (b) two middle *b*-values, (c) middle and highest *b*-values and (d) lowest and highest *b*-values. These double *b-value* data sets were tested using the same classification experiments as described in the previous paragraph.

#### Classification performance metrics

Two performance metrics were used to assess the quality and accuracy for the binary classification experiments. First, the proportion of correctly classified vertices was taken to indicate overall classification accuracy. When averaged across the three subjects it provides an indicator of how well the data with repeated or mixed *b*-values could discriminate tissue in the two ROIs. A one-sided Wilcoxon Rank Sum test across the 21 or 28 binary classification results in the local and HCP data, respectively, tested the hypothesis that classification based on mixed *b*-values outperforms that of the repeated *b-value*. Secondly, an aggregated F1-score, that provides the harmonic mean between precision and recall, was used as a measure of classification accuracy for each ROI. The proportion of true positives in relation to both false positives and false negatives, gives a value between 0–1, with 1 being 100% correct classification, i.e. matching the ground truth labeling [49]. The F1-score was averaged for each ROI across all binary classification tests in which that region was used.

## Results

### Correlation maps

**Fig. 2:**
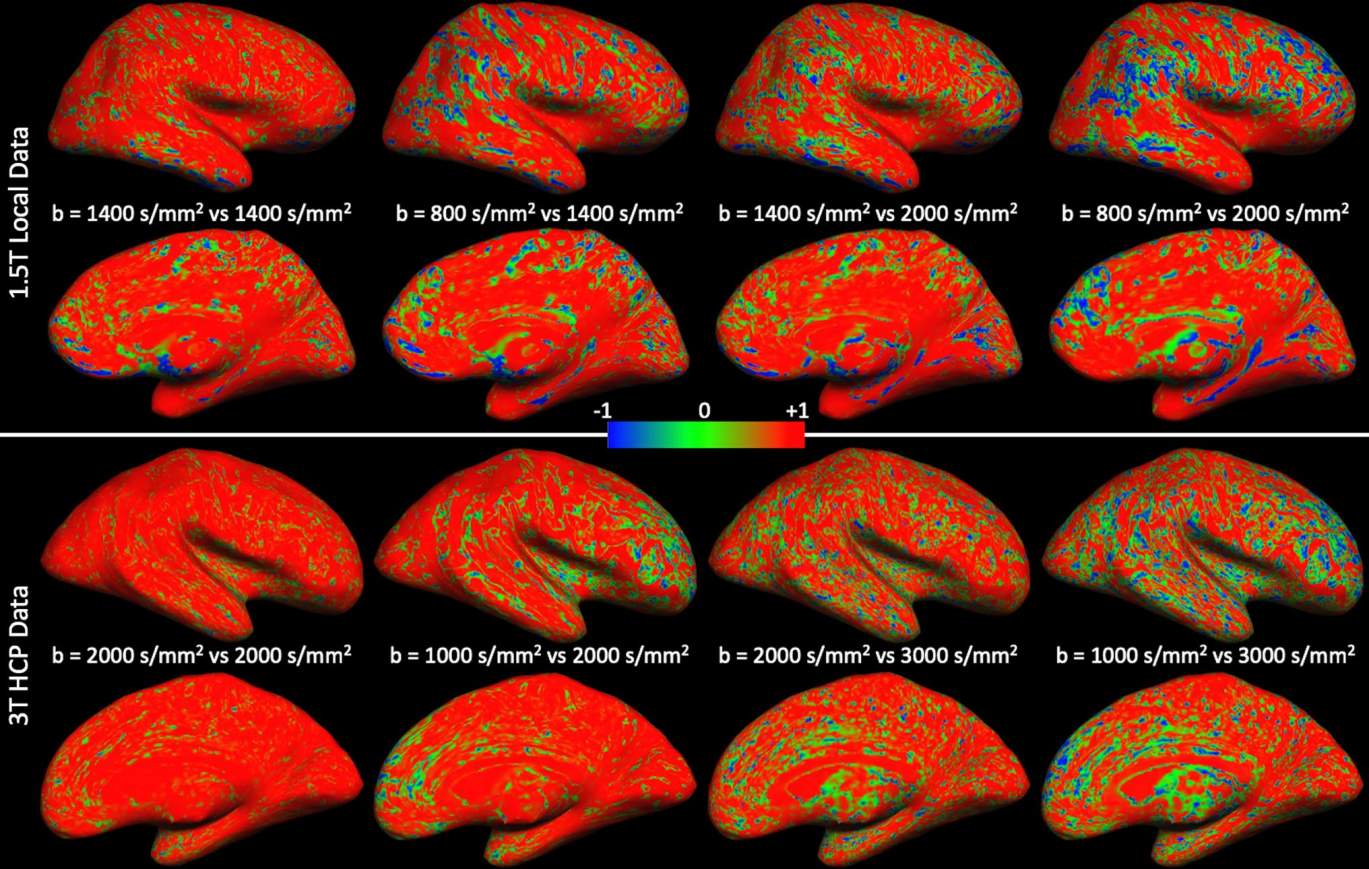
Maps of voxel-wise correlation coefficients between pairs of feature vectors that were obtained from different HARDI data acquisitions for both the local (top) and HCP (bottom) data sets. Note how the correlation drops as the difference in *b-value* for the two acquisitions increases

Figure 2 displays the correlation maps between feature vectors for both the local 1.5T and 3T HCP data. Both data sets showed a similar trend, where the correlation drops as the *b-value* difference increases. These correlation maps suggest that varying information is captured by the different *b*-values and hence using multiple *b*-values should provide a richer feature vector for classification between cortical tissue domains.

The feature vectors from two *b* = 2000 s/mm^2^ subsets were highly correlated through most of the cortical surface, providing confidence that the electrostatic repulsion method that was used to subsample the gradient directions in the HCP data worked adequately.

#### Classification with triple *b*-values

**Fig. 3:**
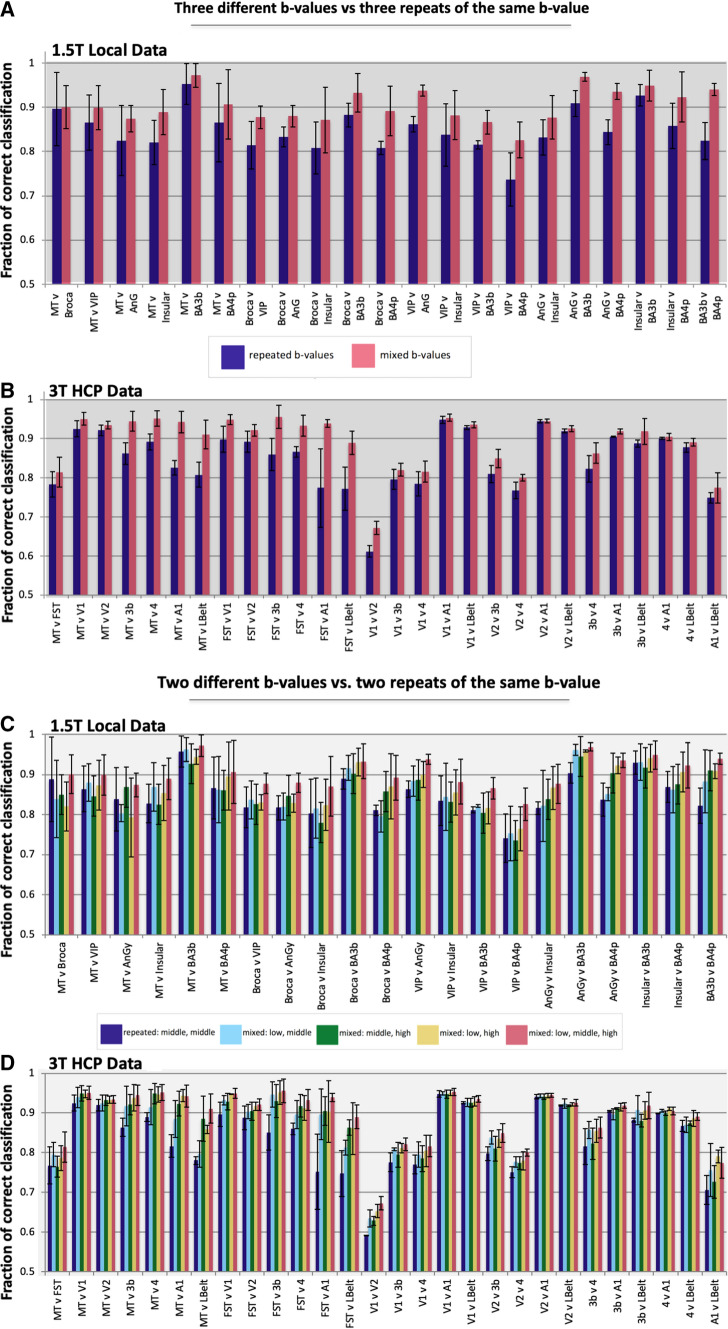
Results of the binary classification experiments for all possible combinations of the ROIs in Fig. 1 for both the local (**a**, **c**) and the HCP (**b**, **d**) data. Combining feature vectors that were obtained from HARDI data sets with three different *b*-values improved the percent of correct classifications as compared to combining feature vectors that were obtained from three repeats of the same *b-value* (**a**, **b**). In general, similar results were obtained when combining features vectors from only two data sets in that combining different *b*-values provides better classification accuracy than repeated acquisition at the same *b*-value (**c**, **d**)

Figure 3 (top) has the performance of the binary classification experiments in data that contained either three different or three times the same *b*-value(s). The height of the bars corresponds to the percent of correctly classified vertices within the two compared regions and averaged across the volunteers. For both the local and the HCP data the feature set obtained from the mixed *b*-values outperformed that of the repeated *b-value* in all of the tests. This supports the hypothesis that measuring diffusion MRI data with multiple *b*-values provides more information to differentiate between cortical areas. On average the improvement in classification accuracy was 5.6% ± 2.6% or 4.6% ± 4.2% across the 21 or 28 binary tests for the local and HCP data, respectively (*p* < 0.001).

**Fig. 4:**
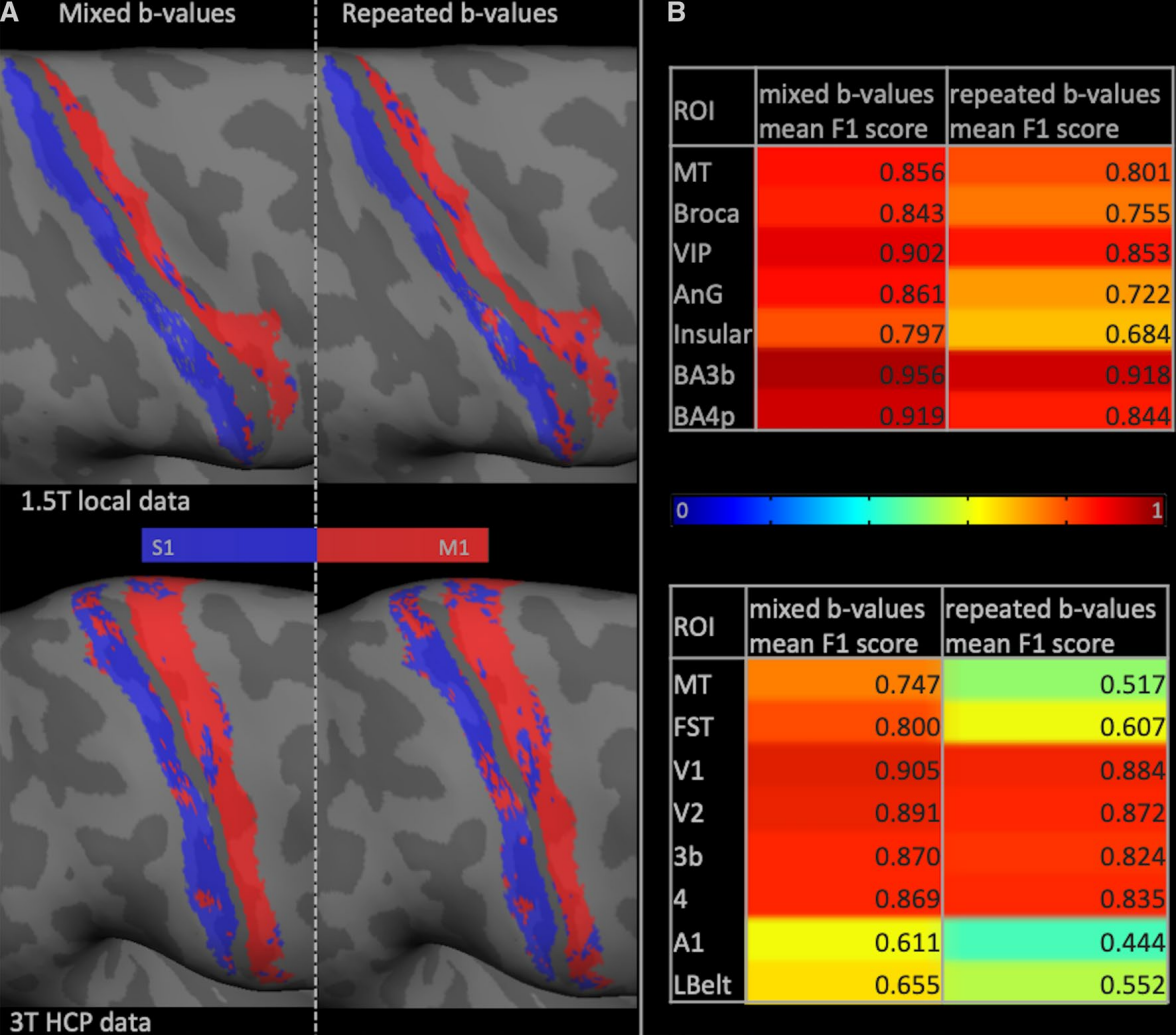
**a** Pictorial representation of a binary classification experiment between the primary motor and sensory cortices for both the local (top) and HCP (bottom) data. **b** Summarized F1 scores for each ROI in Fig. 1 for both the local (top) and the HCP (bottom) data

Figure 4a shows the classification results between areas 3b (S1 region) and 4/4p (M1 region) for a single subject from both the local and HCP data. These regions have widely differing cytoarchitecture [23, 50] but their locations within the central sulcus exhibit low inter-subject variability and hence are likely to be a reliable test-bed. The mixed *b*-values outperformed the repeated *b-value* in both data sets with an average improvement across subjects of 11.6% ± 3.6% and 4.0% ± 1.1%, respectively (Fig. 3). Qualitative assessment (Fig. 4a) revealed that data with mixed *b*-values produced more spatially contiguous results, bearing closer resemblance to the training labels. The summary aggregated F1-score corroborates all above classification results (Fig. 4b).

#### Classification with paired *b*-values

The classification accuracy of the feature sets of paired *b-value* combinations are displayed in Fig. 3 (bottom). For the HCP data, a mix of two different *b*-values outperformed two repeated *b*-values (*b* = 2000 s/mm^2^) in the majority of binary classification tests. The repeated *b-value* data produced higher percent of correct classification than the combination of *b* = 1000/2000 s/mm^2^ only when differentiating area 3b from A1. On average, the combination of *b* = 1000/2000 s/mm^2^ shells performed 3.2% ± 3.1% (*p* < 0.001) better than the repeated *b-value*. The combination of *b* = 2000/3000 s/mm^2^ shells also outperformed the repeated case for the majority of tests (excluding MT vs. FST, V1 vs. A1, V1 vs. Lbelt, V2 vs. Lbelt, and 3b vs. Lbelt) with a mean improvement of 3.4% ± 4.2% (*p* < 0.001). Combining the lowest and highest *b-value* produced a mean classification improvement of 4.6% ± 3.9%, (*p* < 0.001) over repeated *b-value* feature set.

The three possible combinations of two different *b*-values were also compared against each other for the HCP data, yielding mean improvements of 1.2% ± 1.6% and 1.4% ± 1.8% (*p* < 0.001) when comparing classification accuracy for the data with *b* = 1000/3000 s/mm^2^ against that for *b* = 2000/3000 s/mm^2^ and *b* = 1000/2000 s/mm^2^, respectively (Table 1).

**Table 1:**
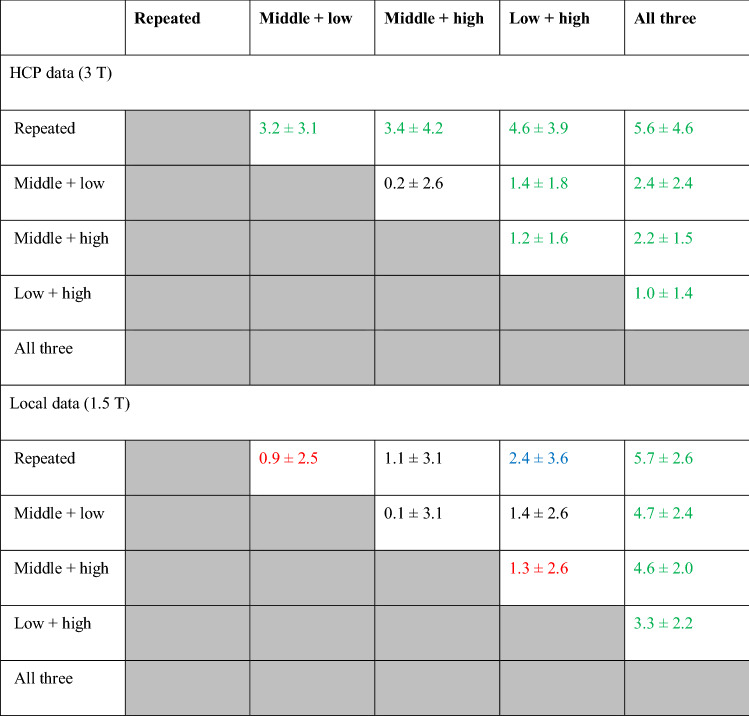
Differences in correct classification rates

The values represent the mean difference ± the standard deviation across all the binary tests in Fig. 3 in percent of correct classification rates. In each entry of the table, a positive value represents the column performing better than the row. The color coding represents the *p*-value provided by the Wilcoxon rank sum test as *α* > 0.05 (black), 0.01 < *α* < 0.05 (red), 0.001 < *α* < 0.01 (blue), *α* < 0.001 (green)

The results from combining all three different *b*-values from the top of Fig. 3 were replicated in the bottom to reinforce that they provide the highest classification accuracy with a mean improvement of 5.6% ± 4.6% over the repeated pair and 1.0–2.4% over the other paired combinations (*p* < 0.001 for all comparisons; Table 1). Combining three *b*-values relies on more data and hence the statistical comparisons are not valid. We provide the three *b-value* case only to ascertain that additional data does indeed lead to an improvement in classification accuracy.

The results for the local 1.5T data exhibit similar trends albeit more variable across classification experiments (Table 1). Also, noteworthy is the finding that the average classification rate with the repeated *b-value* (*b* = 1400 s/mm^2^) was higher in the local 1.5T data than that for the HCP data (*b* = 2000 s/mm^2^). Nevertheless, although the repeated *b-value* combination outperformed one or more of the mixed *b-value* pairs for thirteen of the twenty-one experiments, combining all three *b*-values still always provided the best classification performance (Table 1). The improvement brought on by using all three *b*-values rather than twice middle *b-value* was similar (5.7% ± 2.6%) to the HCP data but larger when comparing against any two different *b*-values (4.7% ± 2.4%, 4.6% ± 2.0% and 3.3% ± 2.2% over the *b* = 800, 400 s/mm^2^, *b* = 1400, 2000 s/mm^2^ and *b* = 800, 2000 s/mm^2^, respectively).

## Discussion

We demonstrated that collecting data with multiple different *b*-values is a better strategy for classification of cortical GM areas than repeating the acquisition with a single *b-value*. This is likely due to the benefit of different *b*-values probing different microstructural properties (i.e. differences in diffusion time or varying lengths of diffusion gradients) and/or because the single most discriminative *b-value* may vary voxel-to-voxel.

In the absence of ground truth microanatomical data, we ran analogous analyses on two different datasets to ensure their results supported each other. Another marker for the robustness of results and methods is the fact that both the subsampled HCP HARDI data and the repeated acquisitions of the same *b-value* data at different time points in the local 1.5T protocol provided highly correlated results (Fig. 2 left column).

The HCP data were collected in a single acquisition with all *b value* shells having the same TE. On the one hand, this is somewhat wasteful of signal-to-noise ratio (SNR), compared to the local 1.5T data, where each *b-value* shell was acquired with the shortest possible TE. On the other hand, if the TEs are not identical for the different *b-value* shells, only mono-exponentials can be fit to the data. To ensure identical treatment of the two data sets we proceeded with mono-exponential fits for both the local and the HCP data.

Although not a formal hypothesis, it is interesting to note that in some cases (e.g. see classification results for area 3b and 4 in Fig. 3) lower correct classification rates were found with the 3T HCP data, despite their arguably better quality. This is likely due to the differences in the protocols (and the subsequently necessary slight deviations in the processing steps). For example, because the HCP protocol acquired a single repeat of each *b-value* shell, we subsampled the 90 diffusion directions into smaller equivalent sets of 30 directions, which likely reduces the specificity of the feature vectors to the local tissue microstructure. Also, while the Connectom scanner is expected to provide higher SNR (i.e. higher main magnetic field and the short TE that is achievable by the strong gradients) [38–40], this SNR was partly traded off for the higher resolution and higher *b*-values (the 3T data has about 59% of the voxel volume and about 1.5 times higher *b*-values compared to the 1.5T data). The improved resolution at the cost of SNR may not provide a universal benefit for all cortical areas.

Nevertheless, on average using all three different *b*-values provided a similar improvement in classification accuracy over using only twice (5.7% ± 2.6% vs. 5.6% ± 4.6% for local and HCP data, respectively) or three times the middle *b-value* (5.6% ± 2.6% vs. 4.6% ± 4.2% for the local and HCP data, respectively). We conclude that using at least two different *b*-values is advantageous for both the 3T HCP and the 1.5T local data sets.

Overall, the correlation maps of Fig. 2 confirmed our hypothesis in both data sets that as the difference in *b-value* increased the feature vectors were increasingly dissimilar. More detailed observations can be made. For example, the local 1.5T data exhibit larger patches of lower correlation across the cortex than do the 3T HCP data when repeated *b*-values are used for classification. It is also noteworthy that while the difference in *b*-values is identical between 800/1400 and 1400/2000 s/mm^2^ as well as between 1000/2000 and 2000/3000 s/mm^2^, when the average *b-value* is higher the correlation drops further. These observations can be attributed, at least partially, to a drop in SNR and voxel size differences, although SNR cannot be the sole factor. The feature vectors are more similar when obtained from two different acquisitions of the same *b-value* (1400 s/mm^2^) than when feature vectors are compared from acquisitions with a *b-value* of 800 or 1400 s/mm^2^, despite the fact that the latter pair (in particular the data with *b* = 800 s/mm^2^) would have a higher SNR.

The cortex in the primary visual area, V1, is known to be thinner than most other cortical areas and is therefore likely to suffer more from partial volume effects and errors in detecting the GM/WM and the GM/pial boundaries. As a consequence, sampling the GM signal from the DWIs in these regions will be less reliable. For these reasons, it was expected that classification accuracy would be significantly lower when one or both of these areas were involved (Fig. 3).

Several avenues are available for further refining the acquisition protocol and the data-driven parcellation method. In future work we plan to systematically and separately vary the diffusion-encoding times and diffusion gradient strength to identify the optimal *b*-values (see for example [51]) in SNR efficient acquisition [52] with spiral [53] rather than echo-planar imaging readouts [54].

## Conclusions

We conclude that, when time is available to collect additional data, varying the *b-value* for the different HARDI data sets is the preferred approach. While acquiring additional data with identical *b-value* increases the SNR, varying the *b-value* will help further improve classification experiments that aim to distinguish cortical GM areas based on their architectonic characteristics.
